# Systemic neuropsychology: toward a contemporary theory of topological functional systems

**DOI:** 10.3389/fpsyt.2026.1745418

**Published:** 2026-02-25

**Authors:** Julián Andrés Guiral

**Affiliations:** 1 School of Arts and Humanities, Instituto de Neuropsicología y Lenguaje (INEL), Medellín, Colombia; 2 School of Arts and Humanities, EAFIT University, Medellín, Colombia

**Keywords:** adaptative systems, anticipatory systems, systemic neuropsychology, topological dynamics, topological functional systems

## Abstract

This article proposes a formalization of psychological functions as Topological Functional Systems, drawing on the historical–cultural tradition and functional systems theory, and integrating them with tools from contemporary topological dynamics. First, it reviews the theoretical foundations that conceive psychological activity as a systemic, dynamic organization oriented toward adaptive outcomes. It then develops a mathematical formalization that accounts for functional invariance under variable modes of execution and network reorganization. On this basis, the article derives clinical implications for neuropsychological assessment and rehabilitation, emphasizing functional reorganization rather than localized deficit. Finally, it discusses the scope and limitations of the model in dialogue with current approaches in neuroscience and neuropsychology.

## 1 Introduction

Contemporary neuroscience is organized, in a cross-cutting manner, around two fundamental organizational principles: the principle of functional segregation, which refers to the relative specialization of brain regions and circuits in specific processing domains, and the principle of integration, which denotes the dynamic and context-dependent coordination of multiple distributed areas for the production of coherent functions at the system level (1). The coexistence of these two principles supports a genuinely systemic conception of the brain, in which interactions among specialized components give rise to emergent properties and patterns of self-organization that cannot be inferred from the isolated analysis of their constituent parts (2–5). Within this framework, psychological functions represent one of the most complex and relevant expressions of such systemic emergence (6–8), insofar as they do not correspond to the activity of discrete modules, but rather to the functional organization of multilevel networks—ranging from local neurophysiological processes to large-scale distributed systems—oriented toward the attainment of adaptive outcomes. From this perspective, psychological functions can be conceptualized as Functional Systems (FS).

A FS is defined as a dynamic organization of the nervous system that is constituted and regulated according to the attainment of a useful adaptive outcome, which acts as the central organizing principle of the system (9, 10). From this perspective, the identity of the system does not reside in a fixed neuronal configuration, but rather in the stability of the outcome to be achieved, allowing the components involved in its realization to vary without the function itself being lost. This idea is articulated with particular clarity in the theory of movement developed by Nikolai Bernstein (11), who demonstrated that the same motor function can be carried out through multiple patterns of muscular activation and distinct kinematic trajectories, while nevertheless preserving the effectiveness of the action. Bernstein emphasized that such variability does not represent a failure of control, but rather a fundamental property of the system, insofar as the function is maintained through the preservation of certain essential functional features of movement, independently of variations in its concrete execution.

This property—the variability of the components that sustain a function while preserving the function itself and its adaptive goal—becomes particularly evident in both development and pathology following brain injury. At the developmental level, the neuroscience of language has shown that the networks supporting linguistic functions do not exhibit a stable organization from early childhood, but instead undergo progressive transformations as the nervous system matures, in a manner consistent with the Vygotskian idea that higher functions emerge through the integration and reorganization of earlier structures (12). Longitudinal and comparative studies indicate that, while certain basic linguistic capacities may rely on early-developing circuits, the consolidation of more complex linguistic functions is associated with the late maturation of specific connections, such as the dorsal pathway including the arcuate fasciculus, whose adult-like organization is not fully present in childhood (13, 14). Complementarily, in the context of brain injury, it has been observed that the execution of the same psychological function can be maintained or recovered through the dynamic reorganization of interactions among neural networks, incorporating new resources and modifying patterns of cooperation among remaining regions, without implying an exact restoration of the pre-injury configuration (15).

This property endows FS with a topological structure, insofar as their identity is not defined by the fixation of components or by specific configurations, but by the preservation of invariant functional relations that allow the same adaptive useful result to be achieved through variable realizations. This conception is implicit in Pyotr Anokhin’s theory of FS, in which the result dynamically organizes and selects the components of the system, and it is expressed with particular clarity in Nikolai Bernstein’s theory of movement, which showed that a single action can remain functionally stable despite variability in its execution patterns. In both cases, what remains is not the fixed composition of the system, but a set of stable functional properties whose preservation confers continuity and identity to the system beyond its particular realizations.

In continuity with this conception, the aim of the present work is to provide a foundation for a formalization of FS as Topological Functional Systems (TFS), in which the functional identity of psychological processes is expressed in terms of dynamic–topological invariants rather than fixed anatomical configurations or local mechanisms. This formalization seeks to rigorously articulate the neuropsychological tradition of functional systems with tools from topological dynamical systems theory, so that variability in components and modes of execution can be understood as a structural property of the system, rather than as a deviation or a failure of control. At the same time, this perspective aims to offer a conceptual framework with direct clinical implications, in which phenomena of development, functional reorganization, and pathology due to brain injury can be interpreted as dynamic transformations of the system that preserve—or alter—its functional invariants. From this standpoint, neuropsychological symptoms are not reduced to deficits localized in isolated components, but are conceptualized as expressions of changes in the trajectories and functional configurations of the system as a whole, thereby opening new avenues for clinical understanding and intervention grounded in systemic, dynamical, and topological principles.

The article is structured into three main sections. The first, “Background and Theoretical Foundations,” presents the foundations of FS and their articulation with the systemic, social, and dynamic conception of psychological functions developed by Vygotski and formalized in the work of Alexander Luria. The second section, “Mathematical Foundations,” introduces a formalization of FS as TFS, articulating the architecture and functionality of FS with tools from topological dynamical systems theory. Finally, the section “Clinical Implications” discusses the scope of this formalization for the neuropsychological understanding of symptoms, proposing a clinical reading centered on the variability of modes of execution and the preservation of functional invariants, with implications for assessment and intervention from a systemic perspective on psychological activity.

In the field of neuropsychology, the present approach seeks to provide continuity and contemporary projection to the scientific program of the historical–cultural tradition, recovering its fundamental orientation according to which psychological and neural activity is constituted through processes of social origin, organized systemically, and dynamically regulated in relation to adaptive outcomes. From this perspective, the proposed Systemic Neuropsychology places TFS at the center of its inquiry as fundamental theoretical units for understanding the organization and variability of psychological functions. Within TFS, the classical foundations of Anokhin’s theory of FS, Vygotski’s historical-cultural conception of psychological processes, and their neuropsychological elaboration in the work of Luria are articulated and integrated with contemporary contributions from neuroscience, dynamical systems theory, and topology. In this sense, the aim of the present work is to establish the theoretical foundations and the mathematical assumptions required to formalize FS as TFS, thereby offering a conceptual framework capable of sustaining clinical and neuropsychological interpretation consistent with the dynamic, variable, and systemic nature of psychological activity.

## 2 Background and theoretical foundations

The systemic conception of the psyche elaborated by Vygotski and Luria is grounded in a historical–cultural perspective that conceives psychological functions as dynamic processes whose genesis cannot be reduced either to the biological or to the individual level. Drawing on the anthropological studies of their time (16), both authors proposed understanding psychological functions as processes that develop across three interdependent domains: (1) the phylogenetic domain, corresponding to biological development from primates to early humans; (2) the historical-genetic domain, corresponding to cultural development encompassing psychological evolution from early humans to contemporary humans; and (3) the ontogenetic domain, which describes individual development from childhood to adulthood, where the biological or phylogenetic domain and the cultural or historical–genetic domain are integrated (17). Taken together, these three domains constitute the essential sources of human psychological development, from which its historical, cultural, and social character is derived—features that establish the necessary conditions for the systemic formation and dynamic organization of psychological activity across ontogeny.

During the ontogenetic development of psychological functions, Vygotski noted that across different stages of development—and in their possible alterations—it is not the function itself that changes, but rather the relations that functions establish among themselves, giving rise to new groupings that did not exist at earlier stages. The formation of these new functional relations into novel groupings is what Vygotski termed Psychological Systems (PS) (18, 19). These systems pass through three constitutive stages: the interpsychological stage, in which the influence of relations with others is operative; the extrapsychological stage, in which this influence is mediated by signs and internalized; and the intrapsychological stage, in which external stimulation—originating from others and mediated by signs—generates intracortical connections that operate within a single functional system, whose organization reflects a dynamic localization in the brain (18, 20). This model overcomes the localizationist and equipotentialist conceptions that dominated understandings of the relationship between the brain and psychological functions in its time.

According to Luria (21), the fundamental error of localizationism and equipotentialism was to conceive psychological processes as immediate products of cerebral structures, without considering the physiological analysis that mediates between these two levels (p. 20). Developing a physiological analysis coherent with the conception of PS requires revisiting the very concept of function, insofar as it constitutes the theoretical foundation of the principle of dynamic localization. In contrast to the classical conception—which defines a function as the activity of a specific organ or tissue, either as a result of the causal role of its mechanisms or of evolutionarily selected effects (22)—a function is now defined as an adaptive activity of the organism oriented toward the accomplishment of a task (9, 21, 22). This activity results from interrelated acts whose execution, in the case of PS, depends on a dynamic and distributed network of connections among highly specialized structures organized across multiple levels of the central nervous system. Within this framework, each function is conceived as a Complex Functional System (CFS) (21), that is, a complex neurophysiological organization that underlies PS and provides the functional scaffolding necessary to understand how they are organized within dynamic brain structures.

The FS, developed by Anokhin in the 1930s, proposes a unit of neurophysiological integration capable of articulating PS with brain structure. Its integrative character lies in its ability to explain the general principles governing any system, among which the adaptive useful result stands out as the central organizing criterion. Unlike the reflex—whose endpoint is the action itself—and classical systemic conceptions based on reciprocal action among components, the FS is defined by the orientation of its processes toward adaptive functional results (23–25). his implies not only interaction among elements, as suggested by reciprocal action, but also an organization directed toward the attainment of biological goals. This approach places FS in alignment with contemporary conceptions of biological systems as open and dynamic structures. As noted by Pessoa (26), adaptive systems transcend the notion of static networks by continuously reorganizing. In this sense, FS anticipate this perspective by conceiving brain activity as an active process of reconfiguration guided by adaptive results.

When the outcome of a functional action is insufficient, the system requires information about that outcome in order to reorganize the distribution of excitations in the appropriate direction. This informational process is referred to, within FS theory, as backward afferentation, which—unlike feedback in mechanical systems—is resolved internally through the system’s own regulatory mechanisms. This afferentation involves a returning sensory flow that is compared with an anticipatory regulatory configuration—the acceptor—and, in the case of discrepancy, initiates a new cycle of functional reorganization (23). In this sense, the FS does not merely correct errors, but adjusts its functional organization as a function of the results obtained, modifying its modes of execution without losing either the identity of the function or the adaptive result that organizes it.

Moreover, because the organism operates within a continuous flow of result attainment, each achieved outcome immediately initiates the preparation of the next. In this process, the resolution of a given result configures the parameters of the subsequent one, generating an anticipatory functional template in memory, referred to as the acceptor. This acceptor fulfills a dual function: on the one hand, it acts as the receiver of the backward afferentation (feedback) generated by the action; on the other, it serves as an anticipatory model of the required result, against which incoming information is compared. When there is a match between the model and the feedback, the result is accepted and the system reorganizes functionally (27). In this way, the FS does not merely respond to past events, but anticipates, infers, and updates its internal organization in accordance with context, the organism’s history, and the expected result, thereby constituting an anticipatory system for the organization of behavior.

The self-organizing character of the FS is expressed through two complementary phases: afferent synthesis and efferent synthesis. Afferent synthesis precedes action and consists of the reciprocal integration of four decisive components: motivation, starting afferentation, contextual afferentation, and memory—understood as historically stabilized functional dispositions rather than stored representations. This interaction reduces the system’s indeterminacy and orients its dynamics toward a specific functional direction. It may involve reverberatory cycles among multiple neuronal elements and culminates in decision making, understood as a reduction of degrees of freedom and the selection of an action as a function of the dominant need, the organism’s experience, and the environmental context. Efferent synthesis, in turn, prepares motor execution through a further reduction of degrees of freedom, adjusting action patterns according to posture, spatial position, and proprioceptive information (23). Taken together, these processes express the system’s capacity to coordinate multiple internal and external sources of information, dynamically reorganizing its functional structure until the programmed useful result is achieved.

The characteristics described make it possible to understand that the useful adaptive result constitutes the organizing axis of the FS. Around this result, the functional architecture outlined above is articulated through a set of interrelated operations—conventionally described as eight moments—including afferent synthesis, decision making, generation of the acceptor of the action result, efferent synthesis, performance of the action, attainment of the result, backward afferentation, and comparison between the obtained result and its anticipated model. This architecture should not be understood as a rigid sequence of discrete states, but rather as an abstract functional organization that is dynamically implemented over time. From this perspective, the FS is not a reactive mechanism, but a dynamic, adaptive, and self-organizing system, capable of anticipating, reorganizing, and adjusting itself on the basis of its own results and the context in which it is embedded.

This architecture of the FS is articulated with the principles of PS in Luria’s conception of CFS. Starting from the premise that psychological activity and brain organization constitute a functional unity, Luria integrates the Vygotskian conception of PS—which emphasizes the social origin, systemic character, and dynamic localization of psychological functions—with Anokhin’s physiological theory of FS, which introduces the principles of adaptation, self-organization, result-oriented regulation, and anticipation. Within this synthesis, PS contribute the historical-cultural and mediated dimension of function, while FS provide the physiological framework that explains its realization and regulation within the central nervous system.

This theoretical convergence makes it possible to understand complex psychological processes not as activities localized in isolated structures, but as distributed functional systems that emerge from the dynamic interaction among cortical, subcortical, and social components. From this integration, Luria formulates three fundamental principles. First, CFS exhibit dynamic localization in the brain, undergoing functional reorganization as a function of the concrete conditions of the act, the task, and the context. Second, they are neither innate nor anatomically preformed, but develop throughout ontogeny through processes of social internalization, reflecting the historical and cultural conditions of the environment. Third, this development is mediated by signs and psychological instruments, with language playing a central role in the reorganization of the system’s functional relations (21). Together, these principles consolidate CFS as a broad explanatory framework in which social genesis and functional regulation converge in the understanding of the brain as a historically organized system.

The conceptions advanced by Vygotsky, Anokhin, and Luria converge on a shared characterization of psychological functions as dynamic functional organizations, historically constituted, goal-oriented, and sustained by variable relations among multiple components. Although this perspective is theoretically robust and clinically fruitful, it calls for a formal framework capable of accounting for functional invariance in the presence of variable realizations, adaptive reorganization across development and following brain injury, and the organizing role of signs and language in the system’s dynamics and topology. In this regard, topological dynamical systems theory offers particularly suitable conceptual and mathematical tools, as it allows the modeling of systems whose identity does not depend on fixed configurations, but rather on the preservation of invariant functional relations under dynamic transformations. The formalization of psychological and functional systems as Topological Functional Systems (TFS) thus constitutes a pathway for rigorously articulating the foundations of the historical–cultural approach with mathematical models capable of coherently supporting both theoretical explanation and the derivation of clinical implications in neuropsychology.

## 3 Mathematical foundation

### 3.1 Conceptual formalization

The characterization of psychological functions as Topological Functional Systems (TFS)—historically constituted, goal-oriented, and sustained by variable functional relations—raises the need for a formal framework capable of rigorously representing the coexistence of functional stability and variability of realizations. Indeed, if the identity of a function does not depend on fixed anatomical components or immutable operational sequences, but rather on the preservation of certain organizing principles that enable the attainment of a useful adaptive result under changing conditions, then static, localizationist, or purely structural models prove insufficient. The very logic of TFS theory calls for a formalization that can describe systems whose organization may transform without loss of identity, and that incorporate anticipation, feedback, and functional reorganization as a function of history and context. In this sense, formalization through tools from topological dynamical systems theory does not constitute an external extrapolation, but rather a natural consequence of the theoretical assumptions developed by Vygotsky, Anokhin, and Luria, insofar as it provides an appropriate language for modeling functional invariance, dynamic reorganization, and historical dependence—core features of psychological activity.

A TFS is proposed as a mathematically parsimonious and theoretically faithful formalization of the central thesis of FS theory: the identity of a function does not reside in a fixed anatomical configuration, but in the stability of the useful adaptive result that organizes the system (9, 20, 21, 25). From this perspective, a TFS is defined over an extended topological state space Z=X×N, where X captures the minimal functional core of the system and N represents the variability of the neural supports that enable multiple realizations of the same function without loss of functional identity.

The introduction of the space N responds to the empirical and theoretical requirement that an TFS can be maintained across variable neural realizations, whether due to development, learning, neurodivergence, or lesion. Rather than assuming predetermined networks, N is conceived as a topological space of high-order network configurations that are dynamically recruited and reorganizable. This conception is consistent with the Vygotskian thesis according to which neurofunctional organization is historically constituted through socially mediated relations by means of signs (29), here understood not as internalized contents but as historically structured dispositions and dynamic constraints shaping the system’s modes of organization for action. Sudakov’s notion of imprints of reality and the Pavlovian concept of the “dynamic stereotype” provide a key physiological justification here: context does not operate merely as a momentary input, but gives rise to functional traces emerging from repeated organism–environment interaction, which persist as historically structured couplings that modulate how the system can reorganize itself for future action (30). These imprints do not constitute representations of environmental states, nor the internalization of contextual information; rather, they act as dynamic constraints on the space of possible functional organizations, conditioning the future selection and stabilization of neural assemblies. In this sense, *N* constitutes the formal locus where context is sedimented as dispositional structure, becoming part of the system’s functional organization without requiring the expansion of *X* to encode or represent contextual openness, as proposed by Pessoa (26).

From this perspective, signs and language—understood as primary sources of historical and social mediation of psychological activity—are not introduced as additional state variables, but rather as semiotic mechanisms of transversal organization of the system. This decision is consistent with the Vygotskian–Lurian tradition, according to which signs do not constitute specific functional contents, but instruments of regulation, anticipation, and reorganization of activity. Within the TFS model, signs modulate the functional core *X* by redefining dominant needs, outcome expectations, and criteria of error; they intervene in *N* as principles of selection, stabilization, and reorganization of neural configurations—in line with the thesis that every neural relation reflects a historically mediated pattern of social interaction (29)—and they act upon the dynamics Φ itself by modifying the system’s modes of evolution through processes of anticipation, regulation, and habit formation. This form of mediation does not presuppose representational encoding of social relations or linguistic meanings. Instead, it operates by reshaping the system’s dispositional landscape, altering the set of functional trajectories that can be dynamically recruited in future situations.

From a mathematical and contemporary neuroscientific perspective, the “higher-order” character of N aligns representationally with developments in higher-order networks, hypergraphs, and simplicial complexes (31, 32), which provide an appropriate language for describing interaction configurations that are not reducible to binary relations but are instead organized into simplices and functional cliques relevant to collective dynamics. This choice does not imply that the topology of N constitutes the invariant of the system; rather, it offers an expressive and reconfiguration-compatible representation capable of capturing how different subjects or conditions—such as childhood and adulthood, lesion and compensation—can implement the same useful outcome through distinct neural assemblies.

Accordingly, the dynamics of the TFS is formulated as a composition of continuous operators that implement the cybernetic cycle described by Anokhin (9)—afferent synthesis, decision making, acceptor of action result, efferent synthesis, action, result, backward afferentation, and comparison—not as discrete phases of the state, but as the internal structure of the dynamic operator. This choice is mathematically conservative and conceptually faithful: it avoids reducing the system to a hybrid automaton and preserves its strict membership within the theory of topological dynamical systems.

In response to objections such as those raised by Pessoa (26) regarding the insufficiency of models centered on static networks or closed state spaces, this formulation offers a precise alternative: contextual dependence is not externalized as a parameter, nor internalized as a representational content, but is historically sedimented as a set of dynamic constraints and dispositional couplings jointly shaping the evolution of *X* and *N*. Through the system’s own history of interaction, context becomes selectable and modifiable as part of the functional organization, without being represented as an explicit state variable. From this perspective, clinical symptoms can be understood as alterations in modes of execution—that is, in functional trajectories and recruitment patterns in *N*—under a relative invariance of the task and the adaptive outcome, with systemic effects arising from the global reorganization of the functional system, an issue that will be developed in greater detail below.

### 3.2 Mathematical formalization

A TFS is formalized as a topological dynamical system (33, 34)


(Z,Φ),

where the state space is defined as the product


Z=X×N,

with X representing the minimal functional state of the system and N the state of network configurations that support its execution.

The minimal functional space is defined as


X:={(n,a,r)},

where n(t) represents the dominant need, understood as the organizing variable that sets the criterion of functional relevance and guides the prioritization of behavior; a(t) represents the anticipatory state of the result (acceptor of action), system-level anticipatory configuration of the expected functional result; and r(t) represents the feedback signal associated with the result effectively attained (or with its internal estimation). From these variables, a functional discrepancy is defined


e(t)=E(a(t),r(t)),

which governs the processes of correction and functional reorganization of the system. Taken together, these variables encode the functional invariants of the TFS without fixing specific anatomical mechanisms, allowing its identity to be expressed as the preservation of the attainment of the useful result under dynamically variable realizations.

The factor N is defined as a topological space of configurations of higher-order neuronal networks that support the execution of the TFS. An element η∈N is interpreted as a continuous descriptor of a higher-order connectivity structure—for example, a hypergraph or a weighted simplicial complex truncated at a maximum order k—which can be represented through a continuous immersion


$$\iota :N\to {\mathbb{R}}^{m},$$

which encodes weights and/or intensities associated with interactions of different orders (edges, triads, 2-simplices, etc.) (32). The evolution of η(t) models the recruitment, plasticity, and reorganization of the network substrate as a function of development, experience, lesion, or neurodivergence, without requiring the existence of pre-specified networks.

The dynamics of the TFS is specified primarily in a cycle-functional manner through a continuous update operator


F:Z→Z,

whose iteration defines a discrete topological dynamical system (Z,F) (33, 34). This operator is factorized as a composition of continuous operators that implement the cybernetic architecture of the functional system:


F=C∘B∘R∘U∘P∘A∘D∘S.

Here, S (afferent synthesis) integrates internal and contextual conditions, updating n and constraining η; D (decision making) selects a functional execution regime; A (acceptor) updates the anticipatory state a; P (efferent synthesis) configures execution parameters over η; U (action) deploys behavior; R (result) determines the attained functional state; B (backward afferentation) updates r with parameters of the obtained result; and C (comparison and correction) computes the discrepancy e = E(a, r) and feeds back onto n and η, including processes of plasticity and functional reorganization. In this formulation, the “components” of the functional system are not introduced as discrete phases of the state, but as the internal structure of the topological dynamics governing the joint evolution of the minimal functional state and the network support.

From a functional standpoint, the operators composing *F* can be grouped into three classes within Φ:

#### 3.2.1 Endogenous operations of prediction and selection

These include S, D, A, and P. These transformations act endogenously on (*X*, *N*), reorganizing the functional state and the network as a function of the system’s own history and current functional organization, without constituting representations of environmental states or relying on internal models of the world.

#### 3.2.2 Coupling with body and world

U and R correspond to the segment of effective interaction with the body and the environment. Topologically, this coupling is incorporated as part of the dynamics in *Z*; conceptually, it represents the “plant–environment” interface through which the system’s functional organization is enacted in the world. In order to preserve the SFT as a formally closed system—closed in the mathematical and dynamical sense, but open in its causal and ecological relations—this coupling is modeled implicitly through the structure of *N* and the mapping that produces the update of *r*. This formal closure does not imply isolation from the environment, nor the existence of internal representations of external states; rather, it ensures that all organism–environment interactions are captured as transformations of the system’s own state space and dynamics.

#### 3.2.3 Feedback and comparison

B and C close the cybernetic cycle, enabling discrepancy-guided correction and adaptive reorganization of the system. Dynamically, these operations are those that confer upon *Φ* its properly cybernetic character, by integrating anticipation, feedback, and functional reorganization within a single topological dynamics.

The proposed formalization seeks to offer an initial mathematical framework for conceiving psychological functions as TFSs whose identity is defined by the stability of a useful adaptive result, rather than by fixed structural configurations. By distinguishing a minimal functional core, a variable network support, and an endogenous cybernetic dynamics, the TFS model makes it possible to describe, in a unified manner, anticipation, feedback, reorganization, and executional variability, which characterize both typical development and the transformations associated with brain injury or neurodivergence. This formalization is not intended to constitute a closed or exhaustive model, but rather a starting point open to refinement, extension, and empirical testing, in dialogue with future developments in both mathematics and neuroscience. Its main value lies in establishing a formal language coherent with the theoretical assumptions of the historical–cultural approach and with the theory of FS, thereby creating the conditions to explore, in the following section, its clinical implications for understanding symptoms, neuropsychological assessment, and functional reorganization.

## 4 Clinical implications

In the effort to construct psychology as a general science grounded in the social, systemic, and dynamic character of psychological functions, Vygotsky proposed a methodological shift of paradigmatic scope. This shift is articulated in two central postulates: first, the replacement of analyses that fragment the system into parts—and thereby lose the emergent properties that only manifest through interaction—with an analysis based on units that preserve the essential properties of the whole; and second, the substitution of the dominant functional or structural analysis with an interfunctional or systemic analysis, oriented toward the study of the relations and connections among functions that are dynamically organized in the brain (18–20). These postulates do not constitute merely an epistemological stance, but rather define a methodological criterion for the scientific study of psychological activity.

From this perspective, the TFS proposal can be understood as a contemporary and formalized extension of that program. At the research level, the foundational characteristics of TFS—the preservation of functional invariants under variable realizations, dynamic reorganization, and contextual dependence—respond directly to the Vygotskian requirement to identify units of analysis that preserve the logic of the system as a whole. In contrast to classical cognitive neuropsychology, where the method of double dissociation has been used to infer stable functional architectures and, in its strong reading, to support a modular ontology of cognition (35–37), the historical–cultural systemic approach—and its formalization as TFS—interprets such dissociations as expressions of differential functional reconfigurations within distributed systems, in which the function is preserved as an adaptive outcome even when modes of execution are altered.

This methodological shift is accompanied by a profound transformation in the conception of the brain. In contrast to modular views, which assume relatively encapsulated and specialized components, contemporary neuroscience has converged on a view of the brain as an integrated dynamic system, in which functional segregation coexists with flexible and context-dependent processes of integration (1, 38). In particular, recent work emphasizes that brain organization is better explained in terms of multiscale adaptive systems, in which functions are sustained by dynamic network configurations that reorganize as a function of task demands, history, and environmental context. This approach explicitly challenges strong modular interpretations of the brain–function relationship, by showing that cognitive processes depend on gradients, interactions among networks, and meso- and microscale structures, rather than on discrete and fixed areas. Accordingly, explanations of brain activity shift toward a pluralistic and integrative framework, in which flexibility and functional overlap reveal the dynamic and contextually dependent nature of neural organization (39).

At the clinical level, this methodological and conceptual shift has direct consequences for the redefinition of both the symptom and neuropsychological rehabilitation. Within the Vygotskian–Lurian tradition, the symptom is not conceived as the direct loss of a function nor as the punctual reflection of a structural lesion, but rather as the manifestation of an alteration in the functional organization of the system, affecting the available modes of execution for achieving an adaptive result (40–43). This conception makes it possible to distinguish between primary symptoms, directly linked to the impairment of specific components or functional trajectories, and systemic symptoms, which emerge as global consequences of the reorganization of the functional system as a whole. It also introduces the notion of functional factors—understood not as modules, but as organizing principles that participate in multiple functions—and situates compensatory processes as active forms of dynamic reorganization, rather than as simple substitutions for lost functions (21, 41, 44).

The clinical approach grounded in TFS, in this sense, is not oriented toward localizing deficits in isolated components, but toward analyzing patterns of functional reorganization, identifying which invariants are preserved, which modes of execution are compromised, and which new trajectories emerge as possible compensations. This perspective is not only consistent with the historical–cultural tradition, but also aligns with the core assumptions of TFS, offering a clinical framework capable of integrating assessment, diagnosis, and intervention within a genuinely systemic conception of the brain and psychological activity.

In a TFS-based clinical framework, the object of description and intervention is not a “function” conceived as an isolable component, but a functional system whose identity is defined by functional invariants (task, criterion of success, adaptive useful result) and whose realization admits multiple modes of execution through the reconfiguration of neural supports and strategies. This shift operationalizes the Vygotskian–Lurian thesis that the deficit is not primarily described as a “loss of function,” but as a reorganization of activity: orientation toward the task or result is preserved, while the dynamic conditions of its realization are altered, giving rise to syndromic profiles that depend on history and context (21, 41, 45, 46).

### 4.1 Clinical unit of analysis in TFS

A TFS-based clinical approach shifts the focus from “functions” conceived as isolable capacities toward units of functional organization defined by (i) invariants (task, criterion of success, and adaptive useful result) and (ii) variability of realization (multiple modes of execution and possible neural supports). This shift is consistent with the contemporary move away from discrete localizations toward circuits and causal networks as explanatory units of symptoms and syndromes: different lesions may converge on similar deficits when they disrupt the same functional circuit, which is precisely the type of “equivalence of realization” that TFS formalizes, rather than treating it as an anomaly of the model (47, 48).

### 4.2 TFS diagnosis: coordinated levels (A–B–C)

#### 4.2.1 Level A: functional invariants

The first diagnostic level makes explicit the core identity of the system: which task/need organizes behavior, what the useful result is (criterion of success), and what the margin of tolerance is (cost/allowable variability before functional collapse). This specification supports a clinical diagnosis oriented toward participation, functioning, and goals, where the criterion of success is not “raising a score” but restoring meaningful functional performance. The tradition associated with Wilson (goal planning) and the evidence synthesized in reviews of cognitive rehabilitation support this view by arguing that the practical unit of intervention and evaluation should be the functional goal (and its attainment), rather than isolated test performance (49).

#### 4.2.2 Level B: loss/degradation of a mode execution

Within the TFS framework, the primary symptom is defined as the restriction or loss of a class of viable dynamic trajectories for achieving an adaptive useful result. This restriction does not imply the disappearance of the function as such, but rather the degradation of a specific mode of execution that previously allowed the function to be carried out in a stable and efficient manner. This mode of execution corresponds to a transfunctional operational principle that participates in the organization of multiple psychological activities. From the TFS perspective, this principle refers to the system’s capacity to sustain a given class of functionally equivalent trajectories associated with a specific type of control (for example, anticipation, sequencing, multimodal integration, or error regulation). The primary symptom emerges when this class of trajectories is lost or becomes unstable, reducing the repertoire of realizations available to the system.

This conception is consistent with contemporary network neuroscience, in which deficits are understood as consequences of perturbing functional circuits that support particular types of control, rather than as the “loss” of isolated cognitive modules. In this sense, approaches such as lesion network mapping provide strong empirical support for models in which different lesions can converge on similar symptoms by affecting common circuits, and in which the same function can be maintained through alternative realizations under conditions of functional reorganization (48).

#### 4.2.3 Level C: reorganization effects (trade-offs)

The third level characterizes the systemic effects of reorganization: costs (time/effort), new contextual dependencies, reduced generalization, fatigue, and compensatory strategies that may appear as “additional deficits” when interpreted from a modular perspective. This level connects both with Luria’s syndromic clinical approach and with contemporary observations of recovery understood as system reconfiguration rather than point-by-point restitution. One advantage of the TFS framework is that it integrates these phenomena as constitutive elements of diagnosis, instead of treating them as noise or nonspecific comorbidity.

At this level, TFS diagnosis characterizes the systemic effects derived from the functional reorganization that the system implements after the loss or degradation of a dominant mode of execution. These effects do not constitute independent deficits, but rather structural costs of the new dynamic regime through which the system attempts to preserve the adaptive useful result. Clinically, they manifest as increased time and effort, greater dependence on contextual cues or external supports, reduced generalization, functional fatigue, and the emergence of specific compensatory strategies. When read through a modular lens, these phenomena may be mistakenly interpreted as “additional deficits” or comorbidities; however, from the TFS perspective, they are understood as necessary consequences of the global reorganization of the system following the degradation of a mode of execution.

This interpretation is fully consistent with Luria’s syndromic clinical approach, in which secondary and tertiary symptoms reflect the redistribution of functional activity, as well as with contemporary rehabilitation frameworks that conceive recovery not as point-by-point restitution of damaged components, but as adaptive reconfiguration of distributed systems, with functional gains accompanied by inevitable trade-offs (50). Within the TFS framework, trade-offs refer to the structural functional costs that emerge when, after the degradation of a mode of execution, the system reorganizes its dynamics to preserve the adaptive useful result, modifying efficiency, flexibility, generalization, or contextual dependence of execution without implying a loss of the function itself.

### 4.3 X = (n, a, r) as clinical variables

The minimal core X = (n, a, r) makes it possible to translate clinical reasoning into observable variables without resorting to a modular catalog of “components.” The dominant need n(t) functions as a clinical prioritization vector (which goals or needs govern the organization of performance), anticipation a(t) reflects the quality of prospective control (preparation, functional set, planning), and the return signal associated with the actually achieved result r(t) captures the quality of monitoring and error-based updating. The discrepancy e(t) = E(a(t), r(t)) operationalizes an interpretable “clinical error.” This articulation allows heterogeneous clinical presentations to be described with a parsimonious language: not damaged “modules,” but dominant patterns of dysregulation (anticipation, monitoring, prioritization) that are also directly translatable into therapeutic targets (anticipatory regulation, improving feedback use, reorganizing goals). This bridge avoids the inferential leap from “dissociation → module,” showing instead how dissociations can emerge from reorganizations of control within distributed systems.

### 4.4 Clinical assessment: from “scores” to a topological–functional profile

The TFS model requires that clinical assessment be structured as a topological–functional profile rather than as an aggregation of isolated scores. This profile integrates, in a coordinated manner: (i) the degree of attainment of the adaptive useful result, (ii) the costs associated with its achievement (time, effort, contextual dependence, fatigue), (iii) the stability, variability, and flexibility of the available execution trajectories, and (iv) the resulting syndromic configuration, understood as the articulation between primary symptoms, systemic effects, and compensatory strategies. This approach is consistent with contemporary goal- and person-centered rehabilitation practices, including methodologies such as Goal Attainment Scaling, which allow the operationalization of individualized functional goal achievement without sacrificing methodological rigor or comparability.

From the TFS perspective, this profile cannot be adequately assessed without recognizing the mediating role of signs and language in the regulation of functional activity. Instructions, self-verbalization, symbolic supports, and external cues are not treated as “methodological aids” that distort measurement, but as structural indicators of the system’s functional regime. Dependence on semiotic mediation, its effectiveness in stabilizing trajectories, and its impact on performance generalization provide key diagnostic information about the organization, costs, and trade-offs of the functional system. In this sense, the contemporary shift toward ecological and digital assessments—together with critiques of clinical inferences based exclusively on decontextualized tests—opens a methodological framework particularly well suited to operationalizing trajectories, costs, and semiotic dependence, precisely the constructs that the TFS model places at the center of clinical analysis (51).

### 4.5 TFS rehabilitation

Within the TFS framework, rehabilitation is defined by two functionally coupled objectives: (i) preserving or restoring the adaptive useful result and (ii) expanding the repertoire of viable trajectories that allow this result to be achieved with a more favorable cost–benefit balance. This formulation is fully consistent with contemporary goal-oriented neuropsychological rehabilitation focused on everyday functioning and on collaboration among patient, family, and therapeutic team, as consistently defended in the clinical literature (49, 50). In contrast to approaches centered on the isolated training of processes, the primary criterion of therapeutic efficacy in the TFS model is not performance on specific tasks, but meaningful functional improvement in real-life contexts.

Operationally, interventions can be organized across three interdependent levels: (I) interventions targeting the functional core X, aimed at recalibrating result anticipation, improving monitoring and error use, and modulating motivational priorities; (II) interventions targeting N, directed at inducing new execution routes, stabilizing functional configurations, and promoting adaptive generalization; and (III) interventions targeting task–context coupling, through the ecological design of activities, difficulty gradients, and contextual manipulation. Across all these levels, signs and language play a central role as instruments of regulation and functional reorganization. Semiotic mediation—through instructions, narratives, self-instructions, explicit rules, and symbolic supports—acts as a clinical operator that enables the modification of priorities, the stabilization of trajectories, the selection of functional configurations, and the reduction of costs without requiring the restoration of damaged components.

From this perspective, language is not conceived as an additional functional domain, but as a transversal instrument of control, anticipation, and compensation, consistent with the Vygotskian–Lurian tradition and with the cybernetic logic of the functional system. TFS rehabilitation therefore does not seek to eliminate semiotic dependence, but to optimize its use, evaluating when it stabilizes functioning, when it introduces undesirable trade-offs, and when it can be stabilized as an endogenous regulatory routine to foster a more autonomous and flexible reorganization of the system.

### 4.6 From the modular to the topological systemic perspective

Rather than treating double dissociation as near-direct evidence for modules, the TFS approach reinterprets it as a possible consequence of: (i) a change in the control regime within X (anticipation/monitoring/prioritization), (ii) alterations in recruitment within N, and (iii) compensatory reorganization accompanied by trade-offs. This position is not merely “philosophical.” There is a substantial classical literature showing that double dissociations can emerge in distributed systems (e.g., connectionist networks) without implying separable components in a strong modular sense, as well as methodological critiques of the inference “dissociation → module” (52). The specific contribution of TFS is to transform this critique into a positive clinical program: it specifies what to measure (invariants, trajectories, costs), how to diagnose (primary vs. systemic effects), and how to intervene (expanding trajectories while preserving the useful result), integrating contemporary circuit/network evidence with the Vygotskian–Lurian systemic interpretation.

### 4.7 From mathematical formalization to clinical practice

The mathematical formalization is not introduced as an ornament, but as a tool to: (i) define invariants and realization equivalences with precision, (ii) model and compare functional regimes (e.g., different X profiles with different costs), and (iii) clinically link symptoms to constraints on trajectories and to changes in recruitment within N. In dialogue with contemporary circuit-based neuroscience, this formalization is compatible with the agenda of moving from “anatomical” descriptions to causal descriptions at the circuit level (*lesion network mapping*), where the clinical hypothesis is formulated as a perturbation of a system regime rather than as damage to a discrete component (48).

In practical terms, the formalism opens three avenues: (1) simulation of intervention scenarios (how changes in anticipation/monitoring or in the stability of network configurations alter functional cost), (2) qualitative prediction of trade-offs (which systemic symptoms are expected given a primary constraint), and (3) the design of dynamics-oriented assessment (which tasks/conditions reveal regime shifts and which measures capture invariants versus costs). This approach also aligns with contemporary trends in neuropsychology toward more integrated and technologically instrumentable frameworks (digital assessment, longitudinal metrics), without collapsing into a reductionism in which “the test is everything” (51).

## 5 Discussion

The present work set out to substantiate a topological formalization of FS that is, at the same time, theoretically congruent with the historical–cultural and neuropsychological tradition inaugurated by Vygotsky and Luria, mathematically consistent with topological dynamics and cybernetics, and clinically pertinent for the understanding and rehabilitation of psychological functions. In this sense, TFS do not constitute a merely descriptive alternative model, but rather an integrative proposal aimed at clarifying the organization, variability, and functional stability of psychological activity under normal conditions, atypical development, brain injury, or psychopathology.

Contemporary neuropsychology has developed multiple frameworks to explain the relationship between brain organization, psychological activity, and clinical symptomatology. In schematic terms, classical and cognitive approaches have shared two central assumptions: (i) a tendency to describe psychological functions as relatively discrete entities—either anatomically localized (53, 54) or implemented as components of cognitive models (35)—and (ii) an interpretation of symptoms as direct expressions of alterations in those components (55). These models have provided valuable methodological tools, particularly for syndromic description and fine-grained deficit analysis, but they show well-documented limitations in accounting for functional reorganization, compensation, and interindividual variability observed both in development and in clinical recovery.

From this perspective, the TFS model aligns with contemporary developments that conceive the brain as a dynamic, distributed, multilevel system, organized simultaneously by principles of integration and segregation (1, 38). Its specificity, however, lies in the fact that it does not adopt these notions merely as empirical descriptions, but articulates them within a functional theory in which the unit of analysis is no longer the “function” as an isolable capacity or the “module” as an encapsulated component, but rather a functional system oriented toward the attainment of a useful adaptive result.

One of the central contributions of the TFS approach is the clarification of the status of functional variability. Rather than interpreting variability as noise, secondary compensation, or failure of control, variability is conceived as a structural property of the system, compatible with the preservation of functional invariants. This idea, already present in Bernstein’s theory of movement and in Anokhin’s theory of functional systems, acquires here an explicit formalization: functional identity is not defined by fixed configurations, but by the preservation of the result across multiple dynamic trajectories.

The introduction of a minimal functional state, organized around need, anticipation, and feedback, further allows for a natural integration of the anticipatory character of psychological activity. At this point, the model enters into dialogue with contemporary predictive approaches—including Bayesian brain models and allostatic formulations (56–58)—but differs from them in a crucial respect: anticipation is not reduced to the abstract minimization of error or surprise, but is organized in relation to historically constituted tasks, needs, and results. This situates prediction within a psychological and functional framework, rather than an exclusively computational one.

At the clinical level, the TFS model proposes a systematic reinterpretation of the neuropsychological symptom. Instead of conceiving it as the loss of a function or an isolable component, the primary symptom is defined as the degradation of a mode of execution, that is, as a restriction of the set of functionally equivalent trajectories available to achieve a useful adaptive result. This degradation does not imply the disappearance of the goal of activity, but rather the loss of stability, efficiency, or viability of a specific class of realizations that previously organized performance. From this initial alteration, systemic symptoms emerge as cascading effects of the global reorganization of the system, including compensatory strategies, functional trade-offs, and new contextual dependencies, which must be interpreted as structural consequences of the new functional regime rather than as additional independent deficits.

This reading allows for an explicit articulation between the syndromic logic developed by Luria and contemporary evidence showing that different lesions can converge on similar functional profiles, and that recovery depends less on anatomical restitution than on the reorganization of networks and strategies. It also provides a coherent framework for goal-oriented rehabilitation, consistent with current models focused on participation, ecological functioning, and quality of life, but with a more explicit theoretical and mathematical grounding regarding what is modified, what is preserved, and how change should be evaluated.

At this point, it is necessary to clarify the status of topology in the TFS proposal. Unlike other contemporary approaches that employ topological tools—such as higher-order networks, simplicial complexes, or derived metrics (31, 32, 59)—primarily for descriptive or analytical purposes on neurobiological data, TFS presuppose topology as a constitutive property of functional organization itself. Topology is not introduced here as an auxiliary language for representing connectivity, but as the mathematical form appropriate for formalizing systems whose identity is defined by relational invariants rather than fixed components.

In this sense, developments such as higher-order networks or simplicial complexes are naturally integrated into the space of functional configurations of the system, but are subsumed within a broader framework in which cybernetic dynamics, anticipation, and semiotic mediation organize activity. The TFS proposal therefore does not merely apply topology to neuroscience, but advances a neuropsychology whose functional ontology is already topological.

This emphasis makes it possible to extend the scope of the model beyond strictly neuropsychological clinical contexts to psychological practice, psychiatry, and education, in coherence with the Vygotskian conception of psychological functions as socially originated and semiotically mediated processes. Within this framework, the mathematical formalization of TFS is not an end in itself, but a tool to clarify concepts, simulate functional dynamics, explore scenarios of reorganization, and ultimately guide clinical and pedagogical decisions in a more systematic manner.

TFS are proposed as an open research program, susceptible to refinement, empirical testing, and expansion, but with a theoretical, mathematical, and clinical foundation sufficiently robust to contribute meaningfully to the contemporary development of a genuinely systemic neuropsychology. Ultimately, we remain convinced that “systems” and “finality” must continue to be the alpha and the omega of our most immediate scientific endeavor.

## References

[1] Tononi G Sporns O Edelman GM . A measure for brain complexity: Relating functional segregation and integration in the nervous system. Neurobiology. (1994) 91. 10.1073/pnas.91.11.5033 PMC43925 8197179

[2] Mitchell M . Complexity: A guided tour. New York: Oxford University Press, Inc (2009). doi: 10.1093/oso/9780195124415.001.0001, PMID:

[3] Pessoa L . The entangled brain. How perception, cognition, and emotion are woven together. Cambridge: The MIT Press (2022). Available online at: http://direct.mit.edu/books/oa-monograph/chapter-pdf/2057574/f000200_9780262372107.pdf (Accessed February 01, 2026).

[4] Safavi S . Brain as a complex system, harnessing systems neuroscience tools & notions for an empirical approach. Tübingen: Max Plack Institute for Biological Cybernetics (2023). doi: 10.15496/publikation-69434.

[5] Tognoli E Kelso JAS . The metastable brain. Neuron. (2014) 81:35–48. doi: 10.1016/j.neuron.2013.12.022, PMID: 24411730 PMC3997258

[6] Kopell NJ Gritton HJ Whittington MA Kramer MA . Beyond the connectome: the dynome. Neuron. (2014) 83:1319. doi: 10.1016/J.NEURON.2014.08.016, PMID: 25233314 PMC4169213

[7] Miller EK Brincat SL Roy JE . Cognition is an emergent property. Curr Opin Behav Sci. (2024) 57:101388. doi: 10.1016/J.COBEHA.2024.101388, PMID: 39651342 PMC11623317

[8] Van Gelder T Port R . It’s about Time: An Overview of the Dynamical Approach to Cognition Mind as Motion: Explorations in the Dynamics of Cognition. Cambridge: MIT Press (1998). doi: 10.7551/mitpress/4622.001.0001

[9] Anokhin PK . *Biology and neurophysiology of the conditioned reflex and its role in adaptive beahvior* (Vol. 3). New York: Pergamon Press Inc (1974).

[10] Anokhin PK Corson SA Corson EOL . Systems analysis of the integrative activity of the neuron. Pavlovian J Biol Science: Off J Pavlovian. (1984) 19:43–101. doi: 10.1007/BF03003132, PMID: 6145141

[11] Bernstein N . The coordination and regulation of movement. London: Pergamon Press Ltd (1967).

[12] Vygotsky LS . The general laws of physical development in the child: the nervous system. In: L. S. Vygotsky’s pedological works, vol. 7. Singapore: Springer (2019). p. 125–42. doi: 10.1007/978-981-15-0528-7_7, PMID: 41728172

[13] Brauer J Anwander A Perani D Friederici AD . Dorsal and ventral pathways in language development. Brain Lang. (2013) 127:289–95. doi: 10.1016/j.bandl.2013.03.001, PMID: 23643035

[14] Friederici AD . Brain structural networks underlying language. Physiol. Rev. (2026) 106:645–674. doi: 10.1152/PHYSREV.00004.2025. PMID: 41213155

[15] Jiang Z Kuhnke P Stockert A Wawrzyniak M Halai A Saur D . Dynamic reorganization of task-related network interactions in post-stroke aphasia recovery. Brain. (2025) 148:3563–75. doi: 10.1093/BRAIN/AWAF036, PMID: 39883566 PMC12493039

[16] Van der Veer R . The anthropological underpinning of vygotsky’s thinking. Stud Soviet Thought. (1991) 42:73–91. 10.1007/BF00818838

[17] Vygotsky LS Luria AR . Studies on the history of behavior: ape, primitive man, and child. New Jersey: Lawrence Erlbaum Associates, Inc (1993). doi: 10.4324/9780203772683, PMID:

[18] Vygotski LS . Sobre los sistemas psicológicos. In: Obras escogidas - I, vol. 1. Madrid: MaChado Grupo de Distribución, S.L (2013). p. 71–93.

[19] Vygotsky LS . Thought and Language (revised and expanded). In: Thought and Language (revised and expanded). Cambridge: The MIT Press (2012). Available online at: https://books.google.com/books/about/Thought_and_Language_revised_and_expande.html?hl=es&id=B9HClB0P6d4C (Accessed January 24, 2026).

[20] Vygotski LS . La psicología y la teoría de la localización de las funciones psíquicas. In: Obras escogidas I, vol. 1. Madrid: MaChado Grupo de Distribuciones, S.L (2013). p. 133–3.

[21] Luria AR . Higher cortical functions in man. 2nd ed. New York: Basic Books, Inc (1980).

[22] Piccinini G . Rethinking biological functions: A goal-contribution approach and its systemic implications (2024). Available online at: https://philosophyofbrains.com/2024/12/08/rethinking-biological-functions.aspx (Accessed October 22, 2025).

[23] Red’ko VG Prokhorov DV Burtsev MS . Theory of functional systems, adaptive critics and neural networks. IEEE International Conference on Neural Networks - Conference Proceedings (2004) 3:1787–1792. doi: 10.1109/IJCNN.2004.1380879, PMID: 41116384

[24] Sudakov KV . Functional systems theory: A new approach to the question of the integration of physiological processes in the body. Neurosci Behav Physilogy. (2004) 34. doi: 10.1023/B:NEAB.0000022636.40887.ef, PMID: 15330289

[25] Sudakov KV . The theory of functional systems: general postulates and principles of dynamic organization (dedicated to the anokhin centenary). Integr Physiol Behav Sci. (1997) 32:392–414. doi: 10.1007/BF02688634, PMID: 9502524

[26] Pessoa L . Beyond networks: Towards adaptive models of biological complexity. Phys Life Rev. (2024) 56:67–81. doi: 10.1016/j.plrev.2025.11.007, PMID: 41351931 PMC13213893

[27] Sudakov KV Anokhin PK . Functional systems theory and the probabilistic prediction of behavior. Neurosci Behav Physiol. (2004) 34:249–52. doi: 10.1023/B:NEAB.0000022638.96382.dd, PMID: 15330291

[28] Vygotski LS . Obras Escogidas III. Historia del desarrollo de las funciones psíquicas superiores. Madrid: MaChado Grupo de Distribución, S.L (2012).

[29] Sudakov KV . Imprints of reality In the systems mechanisms of brain activity. Neurosci Behav Physiol. (2001) 31:7–17. doi: 10.1023/a:1012351708274, PMID: 11766892

[30] Krishnagopal S Bianconi G . Topology and dynamics of higher-order multiplex networks. Chaos Solitons Fractals. (2023) 177. doi: 10.1016/j.chaos.2023.114296, PMID: 41727822

[31] Millán AP Sun H Giambagli L Muolo R Carletti T Torres JJ . Topology shapes dynamics of higher-order networks. Nat Phys. (2025) 21:353–61. doi: 10.1038/s41567-024-02757-w, PMID: 41725453

[32] Auslander J . Minimal flows and their extensions. North-holland mathematics studies (1988). Available online at: http://www.worldcat.org/oclc/476220048 (Accessed January 23, 2026).

[33] de Vries J . Topological dynamical systems: an introduction to the dynamics of continuous mappings. Amsterdam: De Gruyter (2014) p. 498.

[34] Ellis AW Young AW . Human cognitive neuropsychology (Classic edition). 1st ed. New York: Psychology Press (2014). doi: 10.4324/9780203796382, PMID:

[35] Fodor JA . The modularity of mind: an essay on faculty psychology. In: Reasoning. Massachusetts: Cambridge University Press (2012). p. 878–914. doi: 10.1017/cbo9780511814273.046, PMID:

[36] Williams JM Long CJ . Cognitive approaches to neuropsychology. In: Williams JM Long CJ , editors. Cognitive approaches to neuropsychology. New York: Plenum Press (1988). doi: 10.1007/978-1-4684-5577-9, PMID:

[37] Sporns O Tononi G Kötter R . The human connectome: A structural description of the human brain. PloS Comput Biol. (2005) 1:0245–51. doi: 10.1371/journal.pcbi.0010042, PMID: 16201007 PMC1239902

[38] Hayden BY Heilbronner SR Yoo SBM . Rethinking the centrality of brain areas in understanding functional organization. Nat Neurosci. (2025) 2025:1–12. doi: 10.1038/s41593-025-02166-z, PMID: 41436651 PMC13032191

[39] Glozman JM . Quantitative and qualitative integration of lurian procedures. Neuropsychol Rev. (1999) 9. doi: 10.1023/A:1025638903874, PMID: 10468374

[40] Luria AR . Restoration of function after brain injury. London: Pergamon Press (1963).

[41] Luria AR Majovski LV . Basic approaches used in american and soviet clinical neuropsychology. American Psychologist (1977). doi: 10.1037//0003-066x.32.11.959, PMID: 596712

[42] Mikadze YV Ardila A Akhutina TV . A.R. Luria’s Approach to Neuropsychological Assessment and Rehabilitation. Archives of Clin Neuropsychol. (2019) 34:795–802. doi: 10.1093/arclin/acy095, PMID: 30566694

[43] Luria AR . The Functional Organization of the Brain. Sci Am. (1970) 222:66–79. doi: 10.1038/scientificamerican0370-66, PMID: 5413155

[44] Akhutina TV . L.S. Vigotsky y A.R. Luria: la formación de la neuropsicología 1. Rev Española Neuropsicología. (2002) 4:108–29. Available online at: https://dialnet.unirioja.es/servlet/articulo?codigo=2011215 (Accessed February 1, 2026).

[45] Luria AR Simernitskaya EG Tubylevich B . The Structure of psychological processes in relation to cerrebral organization. Neuropsychologia. (1970) 8:13–9. doi: 10.1016/0028-3932(70)90022-9, PMID: 5522545

[46] Joutsa J Corp DT Fox MD . Lesion network mapping for symptom localization: recent developments and future directions. Curr Opin Neurol. (2022) 35:453. doi: 10.1097/WCO.0000000000001085, PMID: 35788098 PMC9724189

[47] van den Heuvel MP Libedinsky I Quiroz Monnens S Repple J Sommer I Cocchi L . Investigating the methodological foundation of lesion network mapping. Nat Neurosci. (2026) 2026:1–11. doi: 10.1038/s41593-025-02196-7, PMID: 41540261 PMC13156034

[48] Wilson BA Evans JJ Keohane C . Cognitive rehabilitation: a goal-planning approach. J Head Trauma Rehabil. (2002) 17:542–55. doi: 10.1097/00001199-200212000-00006, PMID: 12802245

[49] Wilson BA . Goal planning rather than neuropsychological tests should be used to structure and evaluate cognitive rehabilitation. Brain Impairment. (2003) 4:25–30. doi: 10.1375/BRIM.4.1.25.27030, PMID: 33440041

[50] Harris C Tang Y Birnbaum E Cherian C Mendhe D Chen MH . Digital neuropsychology beyond computerized cognitive assessment: applications of novel digital technologies. Arch Clin Neuropsychol. (2024) 39:290. doi: 10.1093/ARCLIN/ACAE016, PMID: 38520381 PMC11485276

[51] Plaut DC . Double dissociation without modularity: evidence from connectionist neuropsychology. J Clin Exp Neuropsychol. (1995) 17:291–321. doi: 10.1080/01688639508405124, PMID: 7629273

[52] Cubelli R . The history of neuropsychology according to norman geschwind: continuity and discontinuity in the development of science. Cortex. (2005) 41:271–4. doi: 10.1016/s0010-9452(08)70913-4, PMID: 15714921

[53] Manning L . Neuropsicología cognitiva: Consideraciones metodológicas. Estudios Psicología. (1990) 43–44:153–68. 10.1080/02109395.1990.10821148

[54] González Nosti M Herrera Gómez E . Evaluación neuropsicológica del lenguaje. Madrid: Editorial Síntesis, S.A (2019).

[55] Friston KJ . Bayesian Dysconnections. In American Journal of Psychiatry. American Psychiatric Association. (2020) 177:1110–2. doi: 10.1176/appi.ajp.2020.20091421, PMID: 33256441

[56] Barrett LF Quigley KS Hamilton P . An active inference theory of allostasis and interoception in depression. Philos Trans R Soc London. Ser B Biol Sci. (2016) 371. doi: 10.1098/RSTB.2016.0011, PMID: 28080969 PMC5062100

[57] Santamaría-García H Migeot J Medel V Hazelton JL Teckentrup V Romero-Ortuno R . Allostatic interoceptive overload across psychiatric and neurological conditions. Biol Psychiatry. (2025) 97:28–40. doi: 10.1016/j.biopsych.2024.06.024, PMID: 38964530 PMC12012852

[58] Expert P Lord LD Kringelbach ML Petri G . Editorial: topological neuroscience. Network Neurosci. (2019) 3:653–5. doi: 10.1162/NETN_E_00096, PMID: 31410371 PMC6663069

